# Assessment of neoadjuvant chemotherapy with docetaxel, cisplatin, and fluorouracil in patients with oral cavity cancer

**DOI:** 10.1002/cam4.5075

**Published:** 2022-07-26

**Authors:** Jin Ye Fu, Xiu Hui Yue, Min Jun Dong, Jiang Li, Chen Ping Zhang

**Affiliations:** ^1^ Department of Oral & Maxillofacial – Head & Neck Oncology, Shanghai Ninth People's Hospital College of Stomatology, Shanghai Jiao Tong University School of Medicine Shanghai China; ^2^ National Clinical Research Center for Oral Diseases & National Center for Stomatology Shanghai China; ^3^ Shanghai Key Laboratory of Stomatology & Shanghai Research Institute of Stomatology Shanghai China; ^4^ Department of Radiology Shanghai Ninth People's Hospital, Shanghai Jiao Tong University School of Medicine Shanghai China; ^5^ Department of Oral Pathology Shanghai Ninth People's Hospital, College of Stomatology, Shanghai Jiao Tong University School of Medicine Shanghai China

**Keywords:** neoadjuvant chemotherapy, oral cavity cancer, progression, response rate, survival, TPF regimen

## Abstract

**Background:**

Chemotherapy with docetaxel, cisplatin, and fluorouracil (TPF) has been studied in patients with head and neck cancer. Its impact on patients with oral cavity cancer was not specified.

**Methods:**

We consecutively reviewed medical files of patients with untreated oral cavity cancer who received neoadjuvant TPF chemotherapy in our department from January 2017 to April 2020. Outcomes included the objective response to TPF chemotherapy, factors associated with the response, and progression and survival in different response groups.

**Results:**

A total of 167 patients were included, with half of stage IV disease. Complete or partial response was observed in 51 patients. A total of 91 patients had stable disease, and 25 patients had progressive disease. The response was not associated with age, sex, anatomic subsite, and the tumor's T stage. It was related with N stage (*p* < 0.001) and clinical stage (*p* = 0.004). Most patients with bulky nodes or nodes with obvious necrosis showed low response or even progressed after neoadjuvant TPF chemotherapy. The planned surgery was conducted in 159 patients. Disease relapse mostly occurred in 2 years after treatment. The 2‐year overall survival and the progression‐free survival were 89.0% and 85.2% for patients with complete or partial response, 62.4% and 55.6% for patients with stable disease, and 12.5% and 4.2% for patients with progressive disease, respectively.

**Conclusions:**

The response of neoadjuvant TPF chemotherapy in patients with oral cavity cancer is related to disease stage, especially the nodal stage. Patients with complete or partial response developed less progression events and better survival.

## Graphical abstract

The response of neoadjuvant TPF chemotherapy in patients with oral cavity cancer was related to the disease stage, especially the nodal stage. Patients with bulky nodes or lymph node with obvious necrosis may have low response. Patients with complete or partial response developed less progression events and better survival.

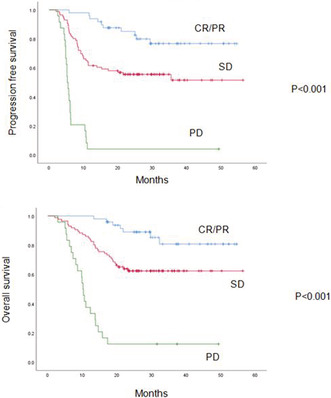

## 1 INTRODUCTION

Oral cavity cancer is common in head and neck, with 377,713 new cases and 177,757 deaths reported in 2020 worldwide.^1^ Typical treatment is surgery with or without adjuvant radiotherapy or chemoradiation.^2^ However, recurrence is common and disease‐related death is significant.^3^ In addition, functional impairment after radical surgery usually leads to a decrease in quality of life. Therefore, investigations on improving outcomes for patients with oral cavity cancer are warranted.

Neoadjuvant chemotherapy has been used in the purpose for decrease the tumor burden to facilitate complete resection and reducing distant metastasis. In head and neck cancers, the docetaxel–cisplatin–fluorouracil (TPF) regimen is conventionally thought to be a standard treatment when induction is considered.^4^, ^5^ Many clinical trials have demonstrated high response rate and improved survival in patients with squamous cell carcinoma of the head and neck who had received TPF therapy.^6^, ^7^, ^8^, ^9^, ^10^, ^11^ Its impact on patients with oral cavity cancer was not specified.

Neoadjuvant TPF chemotherapy has been used in our clinical practice in the wake of its benefits. In this study, we reviewed the medical documents of patients with oral cavity cancer who received TPF treatment preoperatively in our department. Objective response rate and time to progression were evaluated. Overall survival (OS) and progression‐free survival (PFS) were calculated.

## 2 MATERIALS AND METHODS

### 2.1 Patient population

We consecutively reviewed in‐hospital files of patients who were treated in our department from January 2017 to April 2020. The identified cases were patients with treatment‐naive oral cavity cancer and had undergone neoadjuvant chemotherapy, which the following surgery had been planned with curative intent. The chemo‐regimen was confined to TPF, which consisted of docetaxel 75 mg/m^2^ and cisplatin 75 mg/m^2^ on day 1 plus fluorouracil 750 mg/m^2^ on days 1 to 5, every 3 weeks, and for 1 or 2 courses. Dose adjustment was allowed. Pretherapeutic reports of biopsy should be available and confirmed to be oral squamous cell carcinoma. There were no explicit age or tumor stage requirements for inclusion. Patients with metastatic disease at baseline or medical history of malignant tumor were excluded.

This study was approved by the institutional review board of Shanghai Ninth People's Hospital, School of Medicine, Shanghai Jiao Tong University (Approval Number: SH9H‐2020 T129). The informed consent was waived for using medical files, radiological images, and pathological information of the patients. The research was performed in accordance with the provisions of the Declaration of Helsinki.

### 2.2 Data collection

Data collection included patient demographics, smoking and drinking habits, baseline tumor characteristics, radiological images, treatment details, histopathological analysis, and records of postoperative visits in outpatient electronic medical record system. Human papillomavirus (HPV) status was expressed by p16 immunohistochemistry. P16 positive was defined as over 70% of tumor cells showed immunoreactivity. HPV DNA polymerase chain reaction (PCR) was performed in p16 positive cases. The clinical disease stage at baseline was determined by the radiological images and the description of physical examination in medical records according to the eighth edition of the American Joint Committee on Cancer Staging Manual.^12^ Tumor progression and survival data were derived from the outpatient system. Patients after oral and maxillofacial surgery were asked to visit regularly for at least 2 years, preferably 5 years, according to our medical practice. The outpatient system included records of patient follow‐ups, postoperative physical examinations, radiological imagines, and other examines when necessary. The medical files, including the outpatient records, were last reviewed in April 2022.

### 2.3 Response assessment

Response to TPF regimen was determined by comparing radiological images before and after chemotherapy according to RECIST version 1.1.^13^ Clinical descriptions in medical charts were reviewed but not included in the response evaluation. Radiological examinations included computed tomography (CT) and magnetic resonance imaging (MRI) of the oral‐maxillofacial region and the neck. Tumor dimensions on images were measured by two independent radiologists. The longest diameter of primary tumor and short axes of all suspected lymph nodes were recorded. Cervical lymph nodes were given particular concern since oral cavity cancer is prone to have regional lymph node involvement. Nodes with short axis ≥ 15 mm were added to the baseline sum with a maximum of two nodes. Nodes with short axis ≥10 mm to <15 mm and highly suspected were identified as non‐target lesions. The measurements were also recorded for the separate analysis of nodal response.

### 2.4 Statistical analysis

The primary outcome of interest was the response rate of neoadjuvant TPF chemotherapy in patients with oral cavity cancer, which was objectively evaluated by radiological images. Secondary outcomes of interest included factors associated with the response, time to progression after treatment finished, and PFS and OS in different response groups. Progression was defined as locoregional recurrence, distant metastasis, second primary cancer, and death from any cause after treatment finished. Date of first documented progression event was recorded. Time to progression was calculated from the end of definite treatment. Survival data was calculated from the date of treatment starting to the date of either event occurred or last available record before the final review.

The sample size was derived from data availability. It was not calculated upfront because of the observational and retrospective nature of the study. Categorical variables were presented in frequencies and proportions. They were compared by Pearson's chi‐squared (χ^2^) or Fisher's exact tests. Continuous variables were expressed by medians with interquartile range (IQR) and means with standard deviation (SD). They were compared by analysis of variance (ANOVA). The Kaplan–Meier method was used to describe the time‐to‐event data (PFS and OS). Follow‐up duration was calculated by the reverse Kaplan–Meier test. The log‐rank test was used to assess the significance of survival differences among response groups. Hazard ratios (HRs) with 95% confidence interval (CI) were calculated by the Cox‐proportional hazard regression model.

Data analyses were conducted with SPSS version 19.0 for Windows (SPSS Inc.). A two‐sided *p* < 0.05 was considered statistically significant.

## 3 RESULTS

### 3.1 Baseline characteristics

A total of 227 patients with oral cavity cancer had received neoadjuvant TPF chemotherapy during the study period. The assessment was conducted on 167 of them, whose information were eligible for analysis. The main reasons for patient attrition included 43 patients whose radiological images after TPF therapy were not available, another 14 patients with inconsistent radiological methods (i.e., images before and after chemotherapy were CT and MRI, respectively), and 3 patients whose second radiological examination were longer than 42 days after chemotherapy finished. Among patients with complete information, 6 patients had one cycle of the TPF regimen, and 161 patients had two cycles. Both were included in this study.

The patients were predominantly males (73.7%) with median age at diagnosis of 56 years (IQR 47–63). Smoking history was mainly found in male patients. Most patients were p16 negative. Among the six patients with p16 positive, only two had HPV 16/18 PCR positive. Fifty‐two patients were staged of N2 or N3. Over half of the patients were classified as stage IV disease. Eleven patients with stage II had received chemotherapy on the grounds that the waiting time for admission of surgery was long and their clinicians considered applying neoadjuvant chemotherapy during the period. The baseline characteristics of these patients were shown in Table 1.

**TABLE 1:** Baseline characteristics of patients with oral cavity cancer who received neoadjuvant chemotherapy of docetaxel, cisplatin, and fluorouracil (TPF) regimen in the study (*N* = 167)

Variables	No. (%)
Sex	
Men	123 (73.7)
Women	44 (26.3)
Age, years	
Average (SD)	54.8 (11.3)
Median (range)	56 (20–73)
Smoking history	
Current or former	99 (59.3)
Never	68 (40.7)
Drinking history	
Current or former	70 (41.9)
Never	97 (58.1)
Anatomic site	
Tongue	64 (38.3)
Gingiva	38 (22.8)
Bucca	35 (21.0)
Floor of mouth	28 (16.8)
Hard palate	2 (1.2)
Clinical T stage	
T2	34 (20.4)
T3	75 (44.9)
T4	58 (34.7)
Clinical *N* stage	
N0	64 (38.3)
N1	51 (30.5)
N2	47 (28.1)
N3	5 (3.0)
Clinical disease stage	
II	11 (6.6)
III	70 (41.9)
IV	86 (51.5)
p16 status	
Negative	161 (96.4)
Positive	6 (3.6)
TPF cycles	
One	6 (3.6)
Two	161 (96.4)

### 3.2 Response of docetaxel, cisplatin, and fluorouracil regimen

Of the 167 patients, two patients showed no obvious mass with only vague enhancement in postchemo CT images. They were classified as complete response (CR). Among the rest of the patients, 49 (29.3%) achieved partial response (PR), 91 (54.5%) had stable disease (SD), and 25 (15.0%) were observed of progressive disease (PD).

The response was not associated with age, sex, anatomic subsite, and the tumor's T stage. It was related with N stage (*p* < 0.001) and clinical stage (*p* = 0.004) (Table 2). The CR/PR rates were 40.6%, 37.3%, and 11.5% for N0, N1, and N2/3, respectively; and 36.4%, 42.9%, and 19.8% for clinical stage II, III, and IV, respectively.

**TABLE 2:** The association of baseline characteristics with the response of docetaxel, cisplatin, and fluorouracil (TPF) regimen in patients with oral cavity cancer

Variables	CR/PR	SD	PD	Statistical value	*p*‐value
	*n* = 51 (2/49)	*n* = 91	*n* = 25		
Sex					
Men	42	62	19	3.42^a^	0.17
Women	9	29	6		
Age, years					
Average (SD)	54.5 (11.5)	55.1 (11.3)	54.2 (11.2)	0.09^b^	0.92
Median (range)	57 (24–73)	56 (20–73)	56 (34–69)		
Anatomic site					
Tongue	22	34	8	3.99^a^	0.88
Gingiva	9	22	7		
Bucca	10	18	7		
Floor of mouth	10	15	3		
Hard palate	0	2	0		
T stage					
T2	11	20	3	3.04^a^	0.56
T3	26	38	11		
T4	14	33	11		
N stage					
N0	26	34	4	20.07^a^	<0.001
N1	19	26	6		
N2	6	29	12		
N3	0	2	3		
Clinical stage					
II	4	6	1	10.57^b^	0.004
III	30	33	7		
IV	17	52	17		

^a^ Fisher exact chi‐square.

^b^ Analysis of variance.

### 3.3 Better response of primary tumor comparing with regional lymph nodes

When evaluating the responses of neoadjuvant TPF chemotherapy, we observed that the change of primary tumor and regional lymph nodes were not same. Shrinkage of primary tumor was more commonly observed than that of regional lymph nodes. Even in some patients, the primary lesion showed obvious shrunk but the cervical lymph nodes enlarged. Since target lesions in this study were both the tumor in oral cavity and the metastatic lymph nodes in neck, we tried to separately analyze the responses of the primary tumor and the regional lymph node to the TPF regimen in these patients.

For response of primary tumor, 59 (35.3%) patients showed oral lesion reduced at least 30% and 15 (9.0%) patients showed enlargement over 20%. While among 103 patients with clinical N positive, 13 (12.6%) patients showed regional lymph node shrunk at least 30% and 33 (32.0%) patients had enlargement over 20%. The enlargement of lymph nodes occurred more often in originally bulky nodes or nodes with obvious necrosis. The differences between the primary tumor and the regional lymph nodes, of both enlargement and shrinkage, were statistically significant (p values were less than 0.001 for both enlargement and shrinkage).

Although subjective evaluation of clinical response was not taken into consideration in this study, we found in medical records that some patients reported relief of swelling and pain in the primary lesion, improvement of eating, and healing of originally ulcerated tumor surface. They felt that the oral lesion was significantly reduced. But their radiological images did not reach the criteria of CR or PR. Therefore, we defined SD with minor response, which was tumor decreased over 15% but not reached 30% in images. Another 31 (18.6%) patients appeared to reach the SD with minor response of primary tumor. In patients with positive N stage at baseline, few reported relief of symptoms in neck.

Postoperative pathological analysis was reviewed. A total of eight patients did not undergo planned surgery. Complete pathologic remission and microscopic residual of tumor had been observed. Complete pathologic remission was defined as the absence of any tumor cell but leaving keratinous debris in site. Microscopic residual was referred to the presence of scattered foci of a few tumor cells.^14^ There were 8 patients demonstrating pathologic CR and 17 demonstrating microscopic residual in primary tumor site. Their radiological findings of primary lesions were 15 with reduction over 30% and the other 10 with reduction less than 30% or no obvious change in size. Of lymph nodes, complete remission or microscopic residual was found in six lymph nodes, in which five cases had similar appearance in primary lesion. Pathologic CR of overall tumor burden occurred in only two patients of the entire cohort. It is noteworthy that among patients underwent planned surgery, 50 revealed extranodal extension of cervical lymph nodes.

### 3.4 Progression and survival

At the time of last review, 159 patients underwent following surgery, with postoperative radiation or chemoradiation in 137 patients. Patients who did not undergo planned surgery received targeted or immune therapy instead. Thus, they were not followed up. The postoperative treatment was performed according to the NCCN guidelines (the National Comprehensive Cancer Network Clinical Practice Guidelines in Oncology) and at the clinicians' discretion. Radiotherapy was conducted in 6 weeks after surgery with 54 to 60 Gy in total. Concurrent chemoradiotherapy was performed to patients with adverse features, including extranodal extension, close margins, low neck metastasis, and/or invasion of nerve or vascular in pathology. Some patients with stage II or stage III who were considered by their clinicians that the tumor had been radically resected with sufficient margins had received observation and close follow‐up, without radiotherapy or chemoradiotherapy after surgery. The median follow‐up was 30.8 months (IQR 25.6–37.9); 82% of patients were followed for at least 2 years.

**FIGURE 1:**
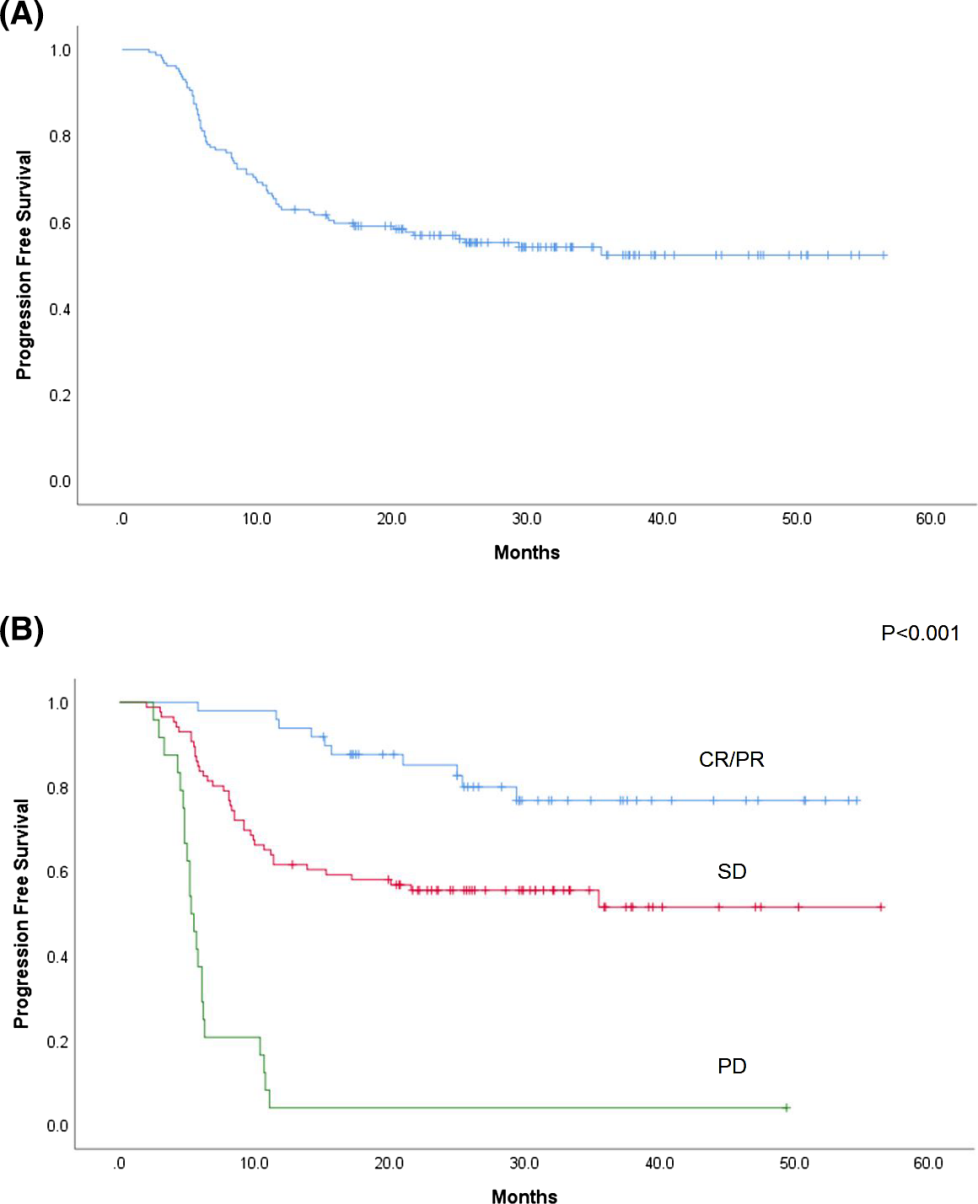
Kaplan–Meier curves for progression‐free survival of the entire cohort (A) and of patients with complete or partial response (CR/PR), with stable disease (SD), and with progressive disease (PD) (B) in patients with oral cavity cancer who received neoadjuvant docetaxel, cisplatin, and fluorouracil chemotherapy.

Overall, progression events occurred in 72 (45.3%) patients: 33 developed locoregional recurrence, 19 had distant metastasis, 9 had both recurrence and metastasis, 4 had second primary tumors, and 7 died of other causes (Table 3). The median time to progression was 3.2 months, ranging from 2 weeks to 30.4 months. Most of the progression events occurred within 2 years after treatment finished (*n* = 70). For patients with CR/PR, the major progression event was locoregional recurrence (7/10). Distant metastasis was rare in this group. For patients with PD, most developed progression events in 6 months after definitive treatment (*n* = 20). Half had distant metastasis. The 2‐year PFS rates were 56.9% for the entire cohort and 85.2%, 55.6%, and 4.2% for CR/PR group, SD group, and PD group, respectively (Figure 1).

**TABLE 3:** Progression after definitive treatment of patients with oral cavity cancer who received neoadjuvant chemotherapy of docetaxel, cisplatin, and fluorouracil (TPF) regimen

Progression after treatment^c^	CR/PR	SD	PD	Statistical value	*p*‐value
	*n* = 49	*n* = 86	*n* = 24		
Time to progression, months					
Average (SD)	12.8 (7.3)	5.1 (5.7)	2.4 (2.6)	13.67^a^	<0.001
Median (range)	10.5 (1.1–25)	3.7 (0.6–30.4)	1.1 (0.5–9)		
Progression events, *n*					
Locoregional	7	16	10	36.99^b^	<0.001
Distant	1	12	6		
Both	0	4	5		
Others	2	7	2		

^a^ Analysis of variance.

^b^ Fisher exact chi‐square.

^c^ A total of eight patients did not undergo surgery and were not followed, two in partial response (PR), five in stable disease (SD), and one in progressive disease (PD). They received targeted or immune therapy instead.

**FIGURE 2:**
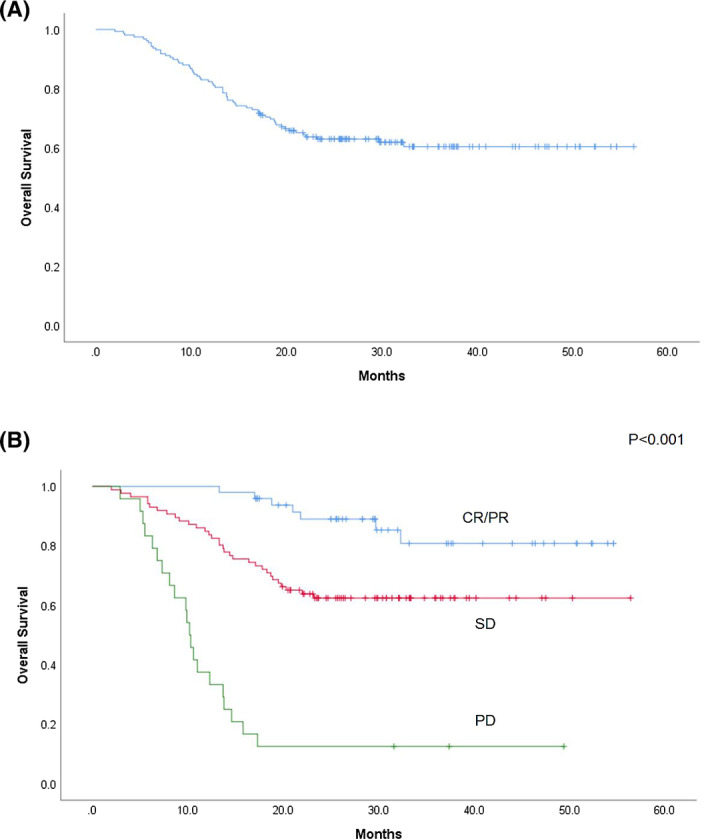
Kaplan–Meier curves for overall survival of the entire cohort (A) and of patients with complete or partial response (CR/PR), with stable disease (SD), and with progressive disease (PD) (B) in patients with oral cavity cancer who received neoadjuvant docetaxel, cisplatin, and fluorouracil chemotherapy.

A total of 60 patients had died before the date of last review. The 2‐year OS rates were 63.0% for the entire cohort and 89.0%, 62.4%, and 12.5% for CR/PR group, SD group, and PD group, respectively (Figure 2). Patients with CR/PR of neoadjuvant TPF chemotherapy resulted in a 70% reduction in the risk of progression (HR 0.28; 95% CI 0.14 to 0.54; *p* < 0.001) and 75% reduction in the risk of death (HR 0.25; 95% CI 0.11 to 0.55; *p* = 0.001) after adjustment for tumor stage.

## 4 DISCUSSION

Oral cavity cancer is a disease mainly treated with surgery. The aim of using neoadjuvant chemotherapy is to improve the outcomes of patients. Although the application of TPF has been studied in patients with head and neck cancers, we focused on its impact on patients with oral cavity cancer.

### 4.1 Reasons for low response rate

The overall response (CR plus PR) rate of TPF in this study was much lower than that previously reported in clinical trials of head and neck cancer.^6^, ^7^, ^8^, ^9^, ^15^ One of the reasons may be the heterogeneous tumor sites. Head and neck cancer commonly consists of cancers of oral cavity, oropharynx, hypopharynx, and larynx. Patients with oral cavity cancer were relatively few in these studies. Oropharyngeal cancer, which was the most common in these trials, is conventionally treated with non‐surgical methods. It is considered to be treatment‐responsive.^16^ For larynx cancer and hypopharynx cancer, TPF therapy showed favorable responses in studies of larynx preservation trials.^17^, ^18^ An earlier study of head and neck cancer, but without patients of oral cavity cancer, showed that the overall response of TPF achieved in 13 of 15 patients.^19^ The responses of TPF regimen in patients with oral cavity cancer were inconsistent in studies. Zhong et al^20^ conducted a phase III randomized trial and reported response rate of 80.6% after two cycles in patients with locally advanced oral cancer. In a recent trial, of which the primary end was mandible preservation rate in patients with oral cavity cancer, the response rate of TPF regimen was 38%.^21^ In another study, which retrospectively evaluated the unresectable oral cavity cancer, the response rate was 28%.^22^

Objective radiologic evaluation without combination of clinical assessment may be another cause. When reviewing medical documents, we found that some patients felt the oral lesions shrunk obviously or clinical examination recorded healing of originally ulcerated tumor surface or significant shrinkage of tumor surface, but the radiological images fail to show such changes or did not reach the criteria of PR. It is difficult to define these subjective responses and hard to evaluate them retrospectively. Also there were some cases missing such information. Thus, we did not combine clinical examination in the response evaluation. In pathological analysis, some patients with complete pathological remission or microscopic residual of tumor did not show CR or PR in radiological images. Therefore, the response of TPF therapy may be underestimated by radiological assessment alone.

### 4.2 Better response in primary tumor than in regional lymph node

In the analysis, we have observed better response of primary tumor than that of regional lymph nodes. Based on the radiological images, the response of primary lesions was almost three times that of metastatic regional lymph nodes. Over half of primary lesions shrank by at least minor response. By pathological analysis, complete remission or microscopic residue of primary tumor appeared in 25 patients. Of lymph nodes, such manifestations appeared in only 5 patients with 6 lymph nodes.

Similarly, some clinical trials have separately reported the response of TPF at the primary site and in the neck. Most revealed higher response of primary tumor than that of regional lymph nodes.^9^, ^23^, ^24^ Researches have shown that therapies designed to treat primary tumors sometimes were less efficacious in treating metastasis lesions because the local tumor microenvironment alters the phenotypic behavior of tumor cells, including response to therapy.^25^ In an animal experiment, it showed that agents slowed the growth of primary tumors but unable to prevent lymphatic metastasis after tumor cells had seeded the lymph node.^26^ In this study, it seemed that primary tumor and metastatic regional lymph nodes may have different response to TPF regimen. Bulky nodes or nodes with obvious necrosis at baseline were inclined to have low response, or even disease progressed, after the treatment.

### 4.3 Response is associated with the clinical stage especially the node status

In our analysis, the response of TPF therapy is associated with the clinical stage of tumor, especially with the lymph node status. The response rates were 42.0% in Stage II/III vs. 19.8% in Stage IV and 39.1% in N0/N1 vs. 11.5% in N2/N3, respectively. The efficacy of TPF therapy in association with tumor stage has been debatable. In a meta‐analysis for oral cavity cancer, patients with cN2 disease showed OS benefit from preoperative chemotherapy, though no significant benefit had been found for the entire cohort.^27^ On the contrary, an Italian study reported lower pathological CR rate in patients with advanced tumor stage. The rates were 41%, 50%, and 9% for stage II, III, and IV, respectively.^28^ In the multivariate analysis for response of induction chemotherapy in patients with head and neck cancer, patients with Stage III achieved more CR comparing with those with stage IV. Disease stage was considered as one of the predictive factors for response.^9^ Indeed, subgroup analysis from trials of head and neck cancer failed to show advantage for the addition of induction TPF in patients with N2/N3 tumor.^10^, ^29^ Although preoperative chemotherapy was designed for reducing local recurrence or distant metastasis, the TPF therapy seemed less responsive in patients with stage IV or N2/N3 oral cavity cancer.

### 4.4 Response can predict the progression and the survival

Response of neoadjuvant TPF is predictive to patient prognosis in this study. Progression events occurred in 20% of patients with CR/PR and the majority was locoregional recurrence. However, almost all patients with PD developed progression events in 1 year after treatment and half was distant metastasis. The 2‐year OS was 89% in CR/PR group compared with 12.5% in PD group. The predictive effect of response to chemotherapy has been reported in previous trials. Patients with clinical CR or pathologic response to induction chemotherapy had better survival than others.^28^, ^30^ In our study, few patients had radiological CR or pathological CR. However, we have observed a strong relationship of the objective response of neoadjuvant TPF therapy with less recurrence or metastasis of patients with oral cavity cancer.

### 4.5 Limitations and highlights

There are some limitations of the study. First is its retrospective nature, of which the selection bias was unavoidable. Patients who did not have radiological images after neoadjuvant chemotherapy were not included in this study. They may have favorable response so that reevaluation was not performed before surgery. In addition, side effect of chemotherapy was not evaluated because of missing data in medical file reviewing. Second, radiological assessment may be difficult in the case of dentures or swallowing artifacts. Two independent radiologists evaluated the images and reevaluated in case of discrepancy. Third, the survival of patients was affected by the treatment they receive after recurrence or metastasis occurred. Some patients participated in targeted therapy or immunotherapy, which may change their survival. Therefore, the occurrence of progression and time to progression may be more objective than survival when evaluated the treatment.

Despite these limitations, there are some highlights of the study. The finding of the relationship between response of TPF chemotherapy and nodal status deserves further study to select patients who would be benefit from the treatment. The better response of the primary lesions than the involving lymph nodes may indicate beneficial in organ preservation practice. Moreover, the predictive effect of response on disease relapse indicted that subsequent treatment could be selected based on the response of neoadjuvant TPF chemotherapy.

## 5 CONCLUSIONS

Our study demonstrates that the response of neoadjuvant TPF chemotherapy in patients with oral cavity cancer is related to disease stage, especially the nodal stage. Patients with bulky nodes or lymph node with obvious necrosis may have low response. In addition, the response of chemotherapy is a prognostic factor that patients with CR/PR developed less progression events, which were mostly locoregional recurrence, and better survival. However, our findings were preliminary and need to be validated in randomized clinical trials.

### AUTHOR CONTRIBUTION

Jin Ye Fu: Conceptualization, data curation, formal analysis, investigation, methodology, validation, writing – original draft, and writing – review and editing. Xiu Hui Yue: Conceptualization, methodology, validation, and writing – review and editing. Min Jun Dong: Conceptualization, methodology, validation, and writing – review and editing. Jiang Li: Conceptualization, methodology, validation, and writing – review and editing. Chen Ping Zhang: Conceptualization, project administration, supervision, and writing – review and editing.

### FUNDING INFORMATION

This work was supported by the Shanghai Clinical Research Center for Oral Diseases under Grant number 19MC1910600 and Shanghai Municipal Key Clinical Specialty under Grant number shslczdzk01601.

## CONFLICT OF INTEREST STATEMENT

The authors have no conflict of interest to declare.

## ETHICS STATEMENT

This study was approved by the institutional review board of Shanghai Ninth People's Hospital, School of Medicine, Shanghai Jiao Tong University. The approval number is SH9H‐2020 T129.

## Data Availability

The data that support the findings of this study are available from the corresponding author upon reasonable request.
